# Effects of Assisted Walking Exercise in Chronic Dependent Ambulatory Stroke Survivors: A Mini-Review

**DOI:** 10.20900/agmr20240007

**Published:** 2024-11-27

**Authors:** Derong Yang, Mustapha Mangdow, Sarah M. Eickmeyer, Wen Liu

**Affiliations:** 1 Department of Physical Therapy, Rehabilitation Science, and Athletic Training, University of Kansas Medical Center, Kansas City, KS 66160, United States; 2 Department of Physical Medicine and Rehabilitation, University of Kansas Medical Center, Kansas City, KS 66160, United States

**Keywords:** stroke, dependent ambulator, walking exercise, chronic stroke rehabilitation

## Abstract

**Background:**

Assisted walking exercise programs are widely recommended in rehabilitation guidelines for stroke survivors. However, most evidence supporting these programs primarily focuses on ambulatory stroke survivors or those dependent ambulatory in acute and subacute stages. There is a notable gap in the application of walking exercise programs for chronic dependent ambulatory stroke survivors despite potential benefits in reducing sedentary behavior and improving rehabilitation outcomes. Thus, this literature review aims to summarize the existing evidence on the feasibility and efficacy of assisted walking exercise programs for chronic stroke survivors who are dependent ambulators.

**Methods:**

Six major databases were searched for clinical trials related to assisted walking exercise and chronic dependent ambulatory stroke.

**Results:**

Seven studies (evidence with low- to moderate-quality) involving 91 chronic dependent ambulatory stroke subjects are included in this review.

**Conclusions:**

These studies indicated that assisted walking exercise is feasible to perform by chronic dependent ambulatory stroke survivors and can induce continued motor recovery and functional improvement. However, the mixed and limited evidence from existing research underscores the need for future high-quality randomized controlled trials with standardized designs and outcome measures to establish evidence-based walking programs for this population.

## INTRODUCTION

Stroke is a leading cause of death and long-term disability worldwide [1]. In the United States alone, there are approximately 795,000 new cases annually [2]. Approximately 57% to 63% of stroke survivors cannot walk independently at stroke onset, and of those, 22% to 50% remain dependent ambulatory even after intensive rehabilitation [3,4]. This loss of the ability to walk independently places a significant burden on the healthcare system and caregivers [5]. Moreover, stroke survivors commonly suffer from cardiovascular diseases (CVDs), such as cardiac diseases, metabolic syndrome, and hypertension [6–11], as well as pulmonary impairment, which limits their exercise tolerance and increases their risk of cardiovascular events, including recurrent stroke [12,13]. These health risks are particularly heightened for chronic dependent ambulatory stroke survivors, who are typically more sedentary than their ambulatory counterparts [14]. Stroke survivors who are more than 3 months post-stroke are generally considered to be in the chronic phase. Beyond 3 months, the rate of natural recovery typically plateaus, and recovery is more dependent on therapeutic interventions rather than spontaneous neuroplastic changes [15]. As such, it is important to create opportunities for chronic dependent ambulatory stroke survivors to be more physically active, promoting potential recovery gains while reducing their risks of CVDs and other health issues.

There is consistent evidence that exercising the lower extremities produces superior cardiovascular fitness gains compared to upper body exercises, as leg muscles not only exert greater strength in pumping venous blood back to the heart but also involve larger muscle groups that increase oxygen demand and heart rate, enhancing cardiovascular efficiency [16,17]. Engaging these muscles improves venous return also helps prevent deep vein thrombosis that may cause sudden death [18,19]. Lower-extremity exercises also stimulate the release of growth hormone and other anabolic hormones, which foster muscle growth, bone density, and overall physical performance [20]. In addition, upright walking exercise may improve pulmonary fitness by increasing oxygen uptake through muscle activation in the trunk and lower limbs [21].

Past studies generated consistent evidence that walking exercise significantly improved mobility (walking speed and tolerance) compared to seated resistance training [22]. Walking exercise is widely recommended in rehabilitation guidelines for stroke survivors because it significantly enhances mobility and overall quality of life [23]. However, the majority of available evidence supporting walking exercise came from clinical trials primarily focused on ambulatory stroke survivors or those in early subacute stages [24–26]. There is a notable gap in the application of walking exercise programs for chronic dependent ambulatory stroke survivors despite the potential benefits in reducing sedentary behavior and improving rehabilitation outcomes for this population. These individuals often face significant barriers to participating in traditional walking exercise programs, typically designed for those who can walk independently or with minimal assistance [27]. Consequently, innovative approaches such as robot-assisted gait training (RAGT), body-weight supported treadmill training (BWSTT), and exoskeletal assistive walking training are being explored to address the unique needs of this population [24,27].

Assisted walking exercises have been used for chronic stroke survivors who are dependent ambulators, but the feasibility and efficacy remain unclear. This literature review aims to investigate the feasibility and effects of walking exercise programs specifically designed for these individuals. By examining recent study outcomes, we seek to highlight the potential benefits of such programs and identify gaps for further research to inform evidence-based interventions for this underserved group.

## MATERIALS AND METHODS

This review focused on rehabilitation of the chronic stroke survivors with dependent ambulation utilizing assisted walking exercise. A combination of controlled Medical Subject Headings (MeSH) and free-text terms relating to the key search terms of “stroke”, “walking”, and “dependent ambulatory” were used to search the following six major databases: PubMed, Google Scholar, EMBASE, CINAHL, Web of Science, and Pedro. The literature search was conducted in November 2024, with search strategies tailored for each database (See Table 1).

We included peer-reviewed research articles that were written in English, published to date, targeted chronic dependent ambulatory stroke survivors (≥3 months post-stroke, ≤3 on FAC [28] or stated that participants were wheelchair-dependent or unable to walk independently), and utilized upright walking exercise. We excluded protocol articles, review articles, and cross-sectional studies.

Two review authors (DY and MM) independently identified all citations, using the predetermined inclusion criteria listed above, discarding those clearly irrelevant. After combining search results into Endnote and removing duplicates, DY and MM independently screened the abstracts of the remaining titles, retaining those that met or potentially met the inclusion criteria. This process was repeated for full-text articles. A third author (WL) was available to enable consensus if there was any disagreement. The reference lists of included studies and relevant reviews identified during the search were also screened.

The studies were reviewed for quality and evidence strength to ensure a rigorous evaluation. The Physiotherapy Evidence Database (PEDro) scale [29], a widely recognized tool, was utilized to assess the methodological quality of clinical trials, focusing on aspects like randomization, blinding, and statistical analysis. Sackett’s Levels of Evidence [30,31], a hierarchical system, were applied to determine the strength of the findings, categorizing evidence based on study design and the quality of the data provided.

## RESULTS

### Included Studies

A total of seven studies involving 91 chronic dependent ambulatory stroke subjects are included in this review (See Table 2).

The studies varied in level of evidence and study quality (see Table 3). Moderate-level evidence and good-quality study design was found in Kelley et al. [33], Kang et al. [40], and Cho et al. [35] due to their randomized controlled trial designs despite modest sample sizes. Low evidence and poor-quality study design characterized Hesse et al. [32], Kawamoto et al. [34], Mazzoleni et al. [36], and Alqahtani et al. [37–39] due to the high risk of bias and small sample sizes in observational or pilot study designs.

#### Type and duration of walking exercise programs

These studies evaluated various walking exercise programs, including robot-assisted gait training (RAGT), body-weight supported treadmill training (BWSTT), and overground gait training (OGT) with assistive devices. The walking exercise programs varied in type and duration: Hesse et al. [32] involved BWSTT and regular physiotherapy, each for 3 weeks with 5 sessions per week, 30–45 minutes per session; Kelley et al. [33] utilized Lokomat RAGT for 60 minutes per session, 5 sessions per week for 8 weeks; Kawamoto et al. [34] focused on OGT using the Hybrid Assistive Limb (HAL) for 20–30 minutes per session, 2 sessions per week for 8 weeks; Cho et al. [35] combined RAGT and conventional physical therapy for 30 minutes per session, 3 sessions per week for 8 weeks; Mazzoleni et al. [36] employed RAGT with sessions varying from 10 to 20, 3–5 days a week; Alqahtani et al. [37–39] used an assistive device and treadmill walking training for 30 minutes per session, 3 sessions per week for 8 weeks; and Kang et al. [40] compared weight support feedback cane OGT to conventional cane OGT, both for 30 minutes per session, 3 sessions per week for 4 weeks.

#### Feasibility of walking exercise programs

High compliance rates and a few adverse events were reported. All studies had high compliance among participants who completed the interventions. Kelley et al. [33] noted minor skin changes in the Lokomat group and a fall in the OGT group. Kelley et al. [33] and Alqahtani et al. [37–39] reported a 9% and 10% withdrawal rate, respectively, due to family issues and other issues unrelated to the interventions. Cho et al. [35] reported a high dropout rate due to health status aggravation, refusal to participate, and adverse dermatological effects, even though the details were not provided.

#### Effects of walking exercise programs

##### Motor function

The studies indicated that various walking exercise programs induce significant gains in motor function in chronic stroke patients with limited ambulation. Hesse et al. [32] showed significant improvements in walking ability (FAC) and walking velocity with BWSTT compared to traditional physiotherapy. Kelley et al. [33] found both Lokomat and OGT effective in improving walking ability and gait parameters, with no significant differences between groups. Kawamoto et al. [34] reported significant enhancements in gait speed, cadence, steps, and balance with Hybrid Assistive Limb (HAL) walking training. Cho et al. [35] highlighted significant improvements in balance and FAC scores with RAGT. Mazzoleni et al. [36] observed notable gains in motor performance, balance, and coordination with RAGT. Alqahtani et al. [37–39] indicated improvements in walking ability and balance with low-intensity aerobic assistive walking exercises. Kang et al. [40] demonstrated greater enhancements in lower limb muscle activity and gait parameters in OGT with weight support feedback cane compared to conventional cane. The observed improvements in motor function across studies suggest that chronic stroke survivors participating in assisted walking exercises may potentially benefit in terms of motor recovery.

##### Aerobic benefits

Alqahtani et al. [37–39] reported significant improvements in cardiovascular risk factors, including decreased glycated hemoglobin (HbA1c), resting heart rate (rHR), systolic blood pressure (SBP), and diastolic blood pressure (DBP). The study also observed enhancements in pulmonary function, indicated by increased forced vital capacity (FVC), and significant improvements in bone health, marked by increased levels of bone biomarkers, including osteocalcin (OC) and carboxy-terminal telopeptide of type I collagen (ICTP).

##### Activities of daily living (ADL) and mental health

Two studies examined the effects of walking exercise programs on daily activities and one study assessed mental health. Kelley et al. [33] reported improvements in physical functional levels and ADL, as measured by Barthel Index (BI), with both Lokomat and OGT. Cho et al. [35] found that RAGT improved ADL scores on the Modified Barthel Index (MBI). Alqahtani et al. [37–39] observed improvements in depression scores, as measured by the Patient Health Questionnaire-9 (PHQ-9).

## DISCUSSION

The findings from the reviewed studies provide promising evidence supporting the feasibility and preliminary efficacy of assisted walking exercise programs for chronic dependent ambulatory stroke survivors. High compliance rates across studies indicate that such interventions are well accepted by participants, with only minor adverse events reported. The improvements in motor function, as indicated by gains in walking ability, balance, and lower limb muscle activity, highlight the potential of these programs to enhance mobility in this population. Additionally, the aerobic benefits, including reductions in cardiovascular risk factors and improvements in pulmonary function, further underscore the value of walking exercise in addressing the broader health concerns associated with chronic stroke. Moreover, the positive impacts on ADL and mental health suggest that these programs can contribute to improved quality of life and psychological well-being for chronic dependent ambulatory stroke survivors.

The outcome measures used across the seven studies varied but primarily focused on motor function, such as walking ability, gait parameters, balance, and muscle activity. Walking ability was commonly assessed with the FAC, 10-meter walk test (10MWT), and 6-minute walk distance (6MWT) to evaluate walking speed and endurance, with the 10MWT and 6MWT being particularly useful for their ease of use and applicability in clinical settings. Balance was frequently evaluated with the Berg Balance Scale (BBS) and Timed Up and Go (TUG) test, both widely recognized for their effectiveness in assessing fall risk and functional stability, which are directly relevant to patients’ daily life activities. Muscle activity was measured using electromyography (EMG) and strength assessments to gauge muscle engagement and recovery. Secondary outcomes included measures of ADL and functional independence, often assessed with the Functional Independence Measure (FIM) and BI, which provide valuable insights into the patients’ capability to perform everyday tasks. Notably, Alqahtani et al. [37–39] included assessments of cardiovascular and pulmonary outcomes, highlighting the potential for walking exercises to improve cardiovascular and pulmonary health, which could mitigate risks of secondary conditions like cardiovascular disease, diabetes, and hypertension [3]. Including such diverse measures offers a holistic view of the effectiveness of walking exercise programs, particularly in promoting functional gains that translate to meaningful improvements in daily life.

We could not directly compare the outcomes between electromechanical-assisted and manual-assisted gait training in chronic dependent ambulatory stroke survivors in the reviewed studies since there is only one case series study of manual-assisted gait training. Although the electromechanical-assisted gait training has not proved its outperformance over manual-assisted gait training [41], it offers substantial benefits in releasing therapists from heavy workload and providing consistent, repetitive, and specialized training [42]. Those advantages are crucial for chronic dependent ambulatory stroke survivors needing long-term rehabilitation to prevent physical deconditioning and comorbidities. However, the high cost and limited availability led to the underrepresentation of electromechanical techniques, including robot-aided training devices, in long-term rehabilitation for chronic dependent ambulatory stroke patients [41]. Moving forward, advancements in mechatronics and AI technology promise future improvements for affordable and smart assistive gait devices suitable for home and community use [43] and consequently call for high-quality and long-term clinical trial studies.

A major limitation of included studies is the lack of high-quality studies that examined the effects of aerobic exercise in stroke survivors with severe impairment [19,21]. The most common form of aerobic exercise after stroke often involves walking [44], but walking is usually considered not suitable for chronic dependent ambulatory stroke survivors. At the chronic stage, stroke survivors with severe impairment frequently encounter a recovery “plateau” [31,45–47], which commonly leads to the termination of rehabilitation treatment, including gait training [48–50]. In addition, cognitive or communication difficulties, lack of motivation or social support, and fear of falling or re-injury further hinder their participation in walking exercises [51]. Furthermore, problems with transportation and lack of suitable assistive devices in community fitness facilities are also barriers for chronic dependent ambulatory stroke survivors to engage in walking exercises.

Another major limitation is the mixed and limited level of evidence of the included seven studies; none of their evidence levels were rated as high. Hesse et al. [32] utilized a single-case A-B-A design, which missed a wash-out period between interventions. Kelley et al. [33] and Kang et al. [40] conducted RCTs with relatively small sample sizes. Kawamoto et al., [34] and Alqahtani et al. [37–39] performed pilot studies with small samples and no control groups. Cho et al. [35] used a crossover RCT but had high dropout rate. Mazzoleni et al. [36]’s observational study had a small sample size. Overall, these studies highlight the need for more extensive, more rigorous trials to validate past findings.

The absence of clearly defined and standardized descriptors for chronic dependent ambulatory stroke survivors has posed significant challenges in selecting and comparing relevant studies. In this focused review, we defined chronic dependent ambulatory stroke survivors as individuals with a FAC score ≤ 3 and post-stroke duration ≥ 3 months. This definition specifically targets a subgroup of stroke survivors who cannot walk independently and rely on wheelchairs for mobility. It also selects the 3-month post-stroke duration as our cutoff for the chronic stage because it represents a critical transition into the chronic phase, during which spontaneous neurological recovery markedly decreases and neuroplasticity becomes less pronounced [42]. Although this timeframe is not widely adopted in stroke rehabilitation research, it remains a topic of ongoing debate. Among the studies reviewed, four [34,35,37,38,40] used 6-month cutoff to define the chronic stage, while three [32,33,36] utilized the 3-month.

The lack of standardized measures for training intensity and varied intervention dosages among the reviewed studies was also a limitation. Kelley et al. [33] conducted 40 1-hour sessions over 8 weeks, classifying their intervention as high intensity. Alqahtani et al. [37–39] described their low-intensity exercise as targeting a heart rate zone of 30% to 40% of the heart rate reserve. The other five studies did not report training intensity. Measuring VO2 max is the gold standard for determining aerobic exercise intensity [52], but it is usually unfeasible in chronic dependent ambulatory stroke survivors [53]. Practical approaches like heart rate monitoring and perceived exertion scales are recommended to ensure consistent training intensity, facilitating better comparison of results and the establishment of best practices [54]. Most walking programs involved sessions three to five times a week, lasting 20 to 60 minutes each, over 4 to 8 weeks, but long-term walking exercise was not studied. While different intervention intensities and dosages have their pros and cons, considering available resources and patient needs, future research may focus on standardizing intensity measurements, exploring different intervention prescriptions, and optimizing training protocols to enhance the effectiveness of walking exercise programs for chronic dependent ambulatory stroke survivors.

## CONCLUSIONS

In conclusion, this mini-review highlights the complexities and challenges of including chronic dependent ambulatory stroke survivors in walking exercise programs. The reviewed studies indicated the feasibility of clinical research on the effect of walking exercise programs in chronic dependent ambulatory stroke survivors. Although the studies indicated the preliminary efficacy of assisted gait training in improving motor function such as walking ability, gait parameters, balance, and muscle activity, the levels of evidence were low to moderate due to small sample sizes and inconsistent methodologies. Additionally, the lack of standardized intervention prescriptions and variability in outcome assessments further complicates the ability to compare and generalize findings. Future studies should aim to standardize design and measures, enhance accessibility, and optimize training protocols better to support the long-term health of chronic dependent ambulatory stroke survivors.

## Figures and Tables

**Table 1.** Search strategy for PubMed (adapted for searching other databases; see Appendix 1).

PubMed
((stroke [MeSH Terms]) OR (Stroke Rehabilitation [MeSH Terms]) OR (Stroke/therapy [MeSH Terms]) OR (Hemiplegia [MeSH Terms]) OR (Stroke) OR (Chronic stroke) OR (stroke/hemiparesis) OR (Stroke Rehabilitation) OR (Stroke therapy) OR (stroke/hemiplegia)) AND ((non-ambulatory) OR (Wheelchair-bound) OR (Dependent ambulator) OR (Immobile) OR (chair bound) OR (Not ambulatory)) AND ((Walking [MeSH Terms]) OR (Gait [MeSH Terms]) OR (Walking exercise) OR (Walking training) OR (Gait exercise) OR (Gait training) OR (Overground walking) OR (Overground gait) OR (Treadmill walking) OR (Treadmill exercise) OR (Treadmill training) OR (Robot-assisted gait training) OR (Robot-assisted walking exercise) OR (Robot-assisted locomotor training) OR (Aerobic walking) OR (robotic-assisted locomotor training))

**Table 2.** Details about studies that met selection criteria.

Author	Sample size^1^	Time since stroke	Level of walking	Study design	Intervention (time per session, sessions per week, weeks)	Main results
Hesse et al. [32]	7	>3 months	FAC ≤ 2	A-B-A case series study	Intervention A: BWSTT (30 min, 5 x, 4 w) Intervention B: Physiotherapy based on the Bobath concept (30 min, 5 x, 4 w)	Significant improvements in gait ability (FAC) and walking velocity during BWSTT compared to regular physiotherapy
Kelley et al. [33]	20	≥3 months	Dependent ambulator	RCT	EXP: RAGT utilized Lokomat (60 min, 5 x, 8 w) CON: OGT (60 min, 5 x, 8 w)	Within-group: Significant gains in LE motor function (FM-LE score) and overall physical functional level (BI) from baseline to post-intervention and baseline to 3-month follow-up within both the RAGT and OGT groups Between-group: no significant differences were found between the RAGT and OGT groups across all outcomes
Kawamoto et al. [34]	8	>6 months	FAC 2–3	Pilot case series study	OGT using Hybrid Assistive Limb (20–30 min, 2 x, 8 w)	Significant gains in walking speed, cadence, number of steps during the 10MWT, and BBS scores
Cho et al. [35]	20	>6 months	FAC < 2	RCT with parallel sequences (AB, BA)	Sequence AB: 4 weeks RAGT using Lokomat + 4 weeks conventional physical therapy (30 min, 3 x, 8 w) Sequence BA: 4 weeks conventional physical therapy + 4 weeks RAGT using Lokomat (30 min, 3 x, 8 w)	Within-group: significant improvements in BBS, MFRT, and MBI in both groups; FAC significantly improved only in the RAGT group Between-group: No significant difference in the BBS, MFRT, MBI, and FAC between groups
Mazzoleni et al. [36]	17	≥3 months	FAC < 3	Multicentric uncontrolled observational retrospective clinical study	RGAT utilizing G-EO System (Ranged from 10 to 20 sessions, 3 or 5 days a week)	Significant gains in global motor performance (FAC and WHS), gait endurance (6MWT), balance and coordination (TUG), lower limb strength (MI), and spasticity (MAS)
Alqahtani et al. [37–39]	9	≥6 months	FAC ≤ 2	Pilot case series study	Assistive treadmill walking training (30 min, 3 x, 8 w)	Significant improvements in HbA1c, rHR, SBP, DBP, bone biomarkers (OC, ICTP), and PHQ-9
Kang et al. [40]	30	>6 months	FAC 2–3	RCT	EXP: OGT with weight support feedback cane (30 min, 3 x, 4 w) CON: OGT with conventional cane (30 min, 3 x, 4 w)	Within-group: Significant improvements were observed within both groups in LE muscle activity and gait parameters, including gait velocity, cadence, affected single-limb support phase, and symmetry index Between-group: The OGT with weight support feedback cane showed significant greater gains in LE muscle activity and gait ability (affected single-limb support phase and symmetry index) compared to OGT with conventional cane

1 The sample sizes listed below are the number of subjects in each study who met the inclusion criteria. BWSTT: body-weight supported treadmill training, FAC: Functional Ambulation Category, RCT: randomized clinical trial, EXP: experimental group, CON: control group, RAGT: robot-assisted gait training, OGT: overground gait training, LE: lower extremity, FM-LE: Fugl-Meyer Lower Extremity scale, BI: Barthel Index, 10MWT: 10-meter walk test, BBS: Berg Balance Scale, MFRT: Modified Functional Reach Test, WHS: Walking Handicap Scale, 6MWT: 6 Minute Walk Test, TUG: Timed Up and Go test, MI: Motricity Index, MAS: Modified Ashworth Scale, HbA1c: Hemoglobin A1c, rHR: resting heart rate, SBP: systolic diastolic blood pressure, DBP: diastolic blood pressure, OC: osteocalcin, ICTP: carboxy-terminal telopeptide of type I collagen, PHQ-9: Patient Health Questionnaire-9.

**Table 3.** Level and quality of evidence supporting assisted walking exercise in chronic dependent ambulatory stroke survivors.

Author	Sackett’s levels of evidence	PEDro items scoring^1^											PEDro total score
		1	2	3	4	5	6	7	8	9	10	11	
Hesse et al. [32]	IV	1	-	-	-	-	-	-	1	1	-	1	3
Kelley et al. [33]	II	1	1	1	1	-	-	1	1	1	1	1	8
Kawamoto et al. [34]	IV	1	-	-	-	-	-	-	1	1	-	1	3
Cho et al. [35]	II	1	1	-	1	-	-	1	1	1	1	1	7
Mazzoleni et al. [36]	IV	1	-	-	-	-	-	-	1	1	-	1	3
Alqahtani et al. [37–39]	IV	1	-	-	-	-	-	-	1	1	-	1	3
Kang et al. [40]	II	1	1	1	1	-	-	1	1	1	1	1	8

1 PEDro items: 1—eligibility criteria were specified; 2—subjects were randomly allocated to groups; 3—allocation was concealed; 4—the groups were similar at baseline regarding the most important prognostic indicators; 5—there was blinding of all subjects; 6—there was blinding of all therapists who administered the therapy; 7—there was blinding of all assessors who measured at least one key outcome; 8—measures of at least one key outcome were obtained from more than 85% of the subjects initially allocated to groups; 9—all subjects for whom outcome measures were available received the treatment or control condition as allocated or, where this was not the case, data for at least one key outcome was analyzed by “intention to treat”; 10—the results of between-group statistical comparisons are reported for at least one key outcome; 11—the study provides both point measures and measures of variability for at least one key outcome.

## Data Availability

No data was generated from the study.

## APPENDIX

**Appendix 1.** Search strategies for other databases (Google Scholar, EMBASE, CINAHL, Web of Science, and PDEro).

Google Scholar
allintitle: chronic stroke walking OR gait OR treadmill OR “robot assisted” -subacute -early -acute -review -arm -upper -hand -finger -protocol -cross-sectional
EMBASE
(‘chronic stroke’/exp OR ‘chronic stroke’) AND (‘walking’/exp OR ‘walking’ OR ‘gait’/exp OR ‘gait’ OR ‘treadmill’/exp OR ‘treadmill’) AND (‘non-ambulatory’ OR ‘dependent ambulatory’ OR ‘dependent ambulator’ OR ‘wheelchair-bound’/exp OR ‘wheelchair-bound’ OR ‘limited ambulation’)
CINAHL
(chronic stroke) AND (walking OR gait OR treadmill) AND (non-ambulatory OR dependent ambulatory OR dependent ambulator OR wheelchair-bound OR limited ambulation)
Web of Science
(chronic stroke) AND (walking OR gait OR treadmill) AND (non-ambulatory OR dependent ambulatory OR dependent ambulator OR wheelchair-bound OR limited ambulation)
PEDro
Simple Search: chronic stroke walking gait treadmill

## ABBREVIATIONS

CVDs: cardiovascular diseases
FAC: Functional Ambulation Category
PEDro scale: Physiotherapy Evidence Database scale
BWSTT: body-weight supported treadmill training
RCT: randomized clinical trial
EXP: experimental group
CON: control group
RAGT: robot-assisted gait training
OGT: overground gait training
LE: lower extremity
FM-LE: Fugl-Meyer Lower Extremity scale
BI: Barthel Index
10MWT: 10-meter walk test
BBS: Berg Balance Scale
MFRT: Modified Functional Reach Test
WHS: Walking Handicap Scale
6MWT: 6 Minute Walk Test
TUG: Timed Up and Go test
MI: Motricity Index
MAS: Modified Ashworth Scale
HbA1c: Hemoglobin A1c
rHR: resting heart rate
SBP: systolic diastolic blood pressure
DBP: diastolic blood pressure
OC: osteocalcin
ICTP: carboxy-terminal telopeptide of type I collagen
PHQ-9: Patient Health Questionnaire-9
ADL: activities of daily living
